# Growth Prior to Thermogenesis for a Quick Fledging of Adélie Penguin Chicks (*Pygoscelis adeliae*)

**DOI:** 10.1371/journal.pone.0074154

**Published:** 2013-09-06

**Authors:** Cyril Dégletagne, Damien Roussel, Jean Louis Rouanet, Fanny Baudimont, Elodie-Marie Moureaux, Steve Harvey, Claude Duchamp, Yvon Le Maho, Mireille Raccurt

**Affiliations:** 1 Laboratoire d’Ecologie des Hydrosystèmes Naturels et Anthropisés, UMR5023, Université Lyon 1, Villeurbanne, France; 2 Department of Physiology, University of Alberta, Edmonton, Canada; 3 Institut Pluridisciplinaire Hubert Curien, Université de Strasbourg, Strasbourg, France; Ecole Normale Supérieure de Lyon, France

## Abstract

The evolutionary trade-off between tissue growth and mature function restricts the post natal development of polar birds. The present study uses an original integrative approach as it includes gene expression, plus biochemical and physiological analysis to investigate how Adélie penguin chicks achieve a rapid growth despite the energetic constraints linked to the cold and the very short breeding season in Antarctica. In pectoralis muscle, the main thermogenic tissue in birds, our data show that the transition from ectothermy to endothermy on Day 15 post- hatching is associated with substantial and coordinated changes in the transcription of key genes. While the early activation of genes controlling cell growth and differentiation (avGHR, avIGF-1R, T3Rβ) is rapidly down-regulated after hatching, the global increase in the relative expression of genes involved in thermoregulation (avUCP, avANT, avLPL) and transcriptional regulation (avPGC1α, avT3Rβ) underlie the muscular acquisition of oxidative metabolism. Adélie chicks only become real endotherms at 15 days of age with the development of an oxidative muscle phenotype and the ability to shiver efficiently. The persistent muscular expression of IGF-1 throughout growth probably acts as a local mediator to adjust muscle size and its oxidative capacity to anticipate the new physiological demands of future Dives in cold water. The up-regulation of T3Rβ mRNA levels suggests that circulating T3 may play an important role in the late maturation of skeletal muscle by reinforcing, at least in part, the paracrine action of IGF-1. From day 30, the metabolic shift from mixed substrate to lipid metabolism, with the markedly increased mRNA levels of muscle avLPL, avANT and avUCP, suggests the late development of a fatty acid-enhanced muscle non-shivering thermogenesis mechanism. This molecular control is the key to this finely-tuned strategy by which the Adélie penguin chick successfully heads for the sea on schedule.

## Introduction

Growth, metabolic intensity, thermoregulation and thermal environment are the key determinants for the full emancipation of offspring. In birds, two strategies, to be born ready (precocial species) or to be born helpless but with a capacity for quick growth (altricial species), govern the acquisition of chick independence and future survival. Yet, growth rate is considerably faster for altricial than for precocial birds. As the development and maturation of organs and tissues are energetically costly, the “tissue allocation hypothesis” [1–6] postulates that the rapid post-natal growth of altricial nestlings precedes the development of thermoregulation. Conversely, the greater thermogenic capacity of precocial chicks is associated with slower growth. There is, therefore, a trade-off between tissue growth and functional maturity that restricts post natal development in most species of birds [7]. This trade-off is particularly challenging for young polar birds. They must grow rapidly and acquire nutritional and thermogenic autonomy while facing very harsh climatic conditions during the short breeding season.

The case of the Adélie penguin chick ( *Pygoscelis* *adeliae* ), traditionally classified as a semi-altricial bird, is of particular interest. Totally dependent on its parents for warmth and food at hatching, it has less than two months to grow, build up energy reserves, moult and develop efficient thermogenic mechanisms before successfully heading out to sea [8,9]. This rapid ontogeny of endothermy could impact and therefore reduce its growth rate. However, the Adélie penguin chick exhibits a high growth constant, as much as 2.2 times higher than the value observed for birds of the same mass living in temperate regions [4,8]. This rapid growth rate is an advantage. It enhances the capacity of young birds to thermoregulate because their surface area/body mass ratio diminishes as chicks get larger, leading to a more favourable heat loss/heat production ratio [10]. In this context, the basic relationship between metabolic maturation and growth rate remains controversial in this species, while most of the information is based on morphological data, energy metabolism and ventilation parameters [7,8]. Adélie penguin chicks therefore appear as useful models to address the trade-off between growth and thermogenesis using biochemical and molecular approaches.

We focused our study on the pectoralis muscle because it represents the engine for the cold-induced thermogenesis of Adélie chicks and its development is essential for their locomotion and future diving ability. In 1983, Ricklefs [11] specified that skeletal muscle is the principal thermogenic organ in the nestling. Moreover, in altricial birds, the onset of endothermy generally correlates with the detection of shivering in the pectoral muscles [12,13]. In a previous study [9], we have shown that in Adélie penguin chicks from 15 to 30 days old the pectoral muscle increases rapidly in mass in comparison to the quadriceps and gastrocnemius and represents a large proportion of the mass of individual chicks. We therefore tested here the hypothesis that the fast development and maturation of pectoralis muscle and the shift from ectothermy to endothermy are both associated with coordinated changes in the transcription of pectoral muscle genes.

Although the function of growth hormone (GH) in growth regulation in birds is not clear, there is evidence, at least in growing chicken, for its growth promoting effect [14,15]. After hatching, GH is crucial for growth by stimulating the production of insulin-like growth factor-I (IGF-1) and interacting profoundly with thyroid hormones (T_3_). Tissue sensitivity depends on the abundance of hormone receptors, thus tissue-specific receptor regulation could enable tissue-specific hormone actions [16]. We therefore tested the muscle expression of these receptors in relation to the growth traits of Adélie chicks from hatching to 60 days old. At the same time, we measured the expression of two mitochondrial proteins involved in thermoregulation, avian uncoupling protein (avUCP), adenine nucleotide translocase (avANT) and their possible transcriptional regulation.

The aim of this study was first, to define the hierarchical transcriptional and biochemical modifications associated with pectoralis muscle development and then to interpret this information in the context of progressive changes in biological and physiological functions. The morphological and enzymatic maturation of growing pectoral muscle was therefore investigated, together with metabolic heat production, the electromyographic activity of shivering and body temperature, which were simultaneously measured at different ambient temperatures in growing Adélie chicks.

This integrative study provides new information on the molecular control of the metabolic/physical transitions from the hatching chick to a mature endothermic Adélie penguin.

## Materials and Methods

All procedures were approved by the Ethics Committee of the Institut Polaire Paul Emile-Victor (IPEV) and by the Polar Environment Committee. They conform to the Code of Ethics of Animal Experimentation in the Antarctic. The Prefect of Terres Australes et Antarctiques Françaises (TAAF) provided the official approval (Authorization N°: MP/11/15/04/09).

### Experimental design and animals

Our study was conducted at Dumont d’Urville, Adélie Land (66°07’ S -140° 00’E) on the Pointe Géologie archipelago where about 34,000 pairs of Adélie penguins ( *Pygoscelis* *adeliae* ) nest every year. During daily surveys from November to February, the hatching and age of nestlings were recorded for 40 nests. From December onwards, the rapid growth of Adélie penguin chicks could be divided into 5 main phases: hatching time (D1), day 7 (D7) characterized by optimal thermal protection of brooding, day 15 (D15) corresponding to partial exposure to cold, day 30 (D30) when chicks are continuously exposed to harsh climatic conditions and finally day 60 (D60), corresponding to their full independence.

The present study was carried out during three summer seasons. Two consecutive summer campaigns were devoted to biochemical and molecular analysis *in vitro*. A total of 24 Adélie chicks were used: six chicks per growth phase (hatching, 7, 15 and 30 days old). Muscles and diverse tissues were weighed and immediately frozen in liquid nitrogen. All samples were stored at -80°C until RNA or protein extractions. A small biopsy of pectoralis muscle was performed on 60 day- old chicks (n=6) for molecular analysis only. A third summer campaign was devoted to measuring metabolic rates and shivering activity *in vivo*. A total of 9-12 chicks per age group (7, 15 and 30 days old) were used. No tissue sampling was performed during this campaign.

### Real time PCR

Total RNAs were extracted from pectoralis muscle samples using TRIzol reagent (Invitrogen). For each sample, 1 µg of total RNA was converted into double-stranded cDNA using 200U of RT-MMLV reverse transcriptase (Invitrogen, Cergy Pontoise, France), 100ng of random primers, 1mM deoxyribonucleotides and 40U RNase inhibitor, according to the manufacturer’s instructions. Real-time PCR was then performed in a MyiQ thermal cycler (Bio-Rad, Marne La Coquette, France) using IQ SYBR Green Supermix (Bio-Rad) to measure the expression of genes involved in growth (avGHR, avIGF-1R and avIGF-1), in thermoregulation [avian uncoupling protein (avUCP), adenine nucleotide translocase (avANT), lipoprotein lipase (avLPL)] and transcriptional regulation [peroxisome proliferator-activated receptor-γ co-activator-1α (avPGC1α) and T_3_ receptor β (avT3Rβ)]. Primers were defined according to chicken (*Gallus gallus*) gene sequences and synthesized by Invitrogen - Life Technologies (Cergy Pontoise, France) (Table 1). Specific amplification of the targeted cDNAs was confirmed by sequencing, which showed more than 90% identity compared to chicken gene sequences.

**Table 1:** Primers defined according to chicken (*Gallus gallus*) gene sequences.

**Genes**	**5’ primers and 3’ primers**	**Fragment size (bp)**	**GenBank accession number**
avGHR	Forward : CAACGATGACTTGTGGGTTG Reverse : CTTCACTCAGGAGCCTGTCA	95	NM_001001293
avIGF-IR	Forward : CGTGGGGACCTCAAAAGTTA Reverse : CCATCCCATCAGCAATCTCT	121	NM_205032
IGF-I	Forward : GCTGAGCTGGTTGATGCTCT Reverse : CACGTACAGAGCGTGCAGAT	202	NM_001004584
avT3Rβ	Forward : AGCAGCAGTTCGCTATGACC Reverse : ACAGCCAGTAGTGCGACAGG	484	NM_205447
avUCP	Forward : ACAACGTCCCCTGCCACTTC Reverse : TTCATGTACCGCGTCTTCAC	299	AY_592972
avANT	Forward : GATGATTGCTCAGACGGTCA Reverse : GCAATCTTCCTCCAGCAGTC	144	XM_001235391
β-actin	Forward : GACGAGGCCCAGAGCAAGAGA Reverse : GGGTGTTGAAGGTCTCAAACA	225	NM_031144
PGC-1α	Forward : GACTCAGGTGTCAATGGAAGTG Reverse : ATCAGAACAAGCCCTGTGGT	271	NM_001006457
avLPL	Forward : GACGGTGACAGGAATGTAGT Reverse : GAGTCAATGAAGAGAGATGGATGG	584	NM_205282

The following qPCR conditions were used: 3 min at 95°C, followed by 40 cycles of denaturation for 10 s at 95°C and annealing/extension for 1 min 30 s at 60°C, according to the manufacturer’s instructions. All samples were measured in duplicate along with dilutions of known amounts of target sequence to quantify the initial cDNA copy number (Concentration = Efficiency^ΔCt^). The results are expressed as the ratio of the target gene over β-actin mRNA concentration, which was checked to ensure non-significant variation between the different groups of cDNAs.

### Western Blotting

Approximately 50 mg of frozen muscle tissue were homogenized in 5% sorbitol, 25mM histidine, 50mM KCl buffer containing 10µL/mL of protease inhibitor with a TissueLyser LT (Qiagen). Homogenates were centrifuged at 1,000 *g* for 5 min at 4°C. Protein concentrations were measured in the supernatant using the Pierce BCA Protein Assay Kit (Thermo Scientific), with bovine serum albumin as standard. 30µg of proteins with Laemmli buffer (Tris/HCl 0.5M pH 6.8, SDS 10%, DTE 0.5M) were boiled at 100°C for 5 min, separated by SDS polyacrylamide gel electrophoresis and transferred to a PVDF membrane. Membranes were blocked for 1 h in TBS blocking buffer containing 5% milk and incubated overnight at 4°C with a goat anti-avANT antibody (ANT SC9299, Santa Cruz, 1:1,000) or a mouse anti-chicken GH receptor antibody (GHR Chs 17, 1: 2,000). Antibody binding was detected using anti-goat peroxidase antibody produced in rabbit (Sigma, 1:10,000) or anti mouse peroxidase antibody produced in goat (Sigma, 1:10,000), respectively, and an enhanced chemiluminescence (ECL Plus) detection kit (Amersham, UK). Protein size was determined using molecular-mass standards (Euromedex, France). Quantification of autoradiography was performed by scanning densitometry. Blots were stripped at 60°C for 30 min in buffer containing 100 mM β-mercaptoethanol, 2% SDS, 62.5 mM Tris/HCl pH 6.8 and rinsed extensively before being re-probed with anti-β-actin for the loading control.

### Metabolic rate and shivering measurements

Using an open-circuit temperature-controlled respiratory chamber, as previously described [17,18], the metabolic rate of 7, 15 and 30 day- old chicks was measured by indirect calorimetry. Their body (stomach) temperature was continuously monitored with copper-constantan thermocouples connected to a Leeds and Northrup 12-channel recording potentiometer (accuracy ± 0.2°C). Due to the electromagnetic activity of the scientific station, shivering was not quantified by electromyography (EMG) but by accelerometry through whole body muscle tremor. A triaxial accelerometer (Entran FEGAS-192 model) fixed on the chick’s back was used. This accelerometry method was tested on ducklings in our lab before being used on penguin chicks in the Antarctic station. To ensure that accelerometry was a suitable technique to record shivering activity, we simultaneously used EMG and accelerometry in ducklings exposed to thermoneutral (25°C) or cold (12°C) temperatures. We found that EMG and accelerometry signals detected muscle activity at the same time (Figure S1), showing that accelerometry is as sensitive as EMG for detecting shivering activity. Data were recorded and analysed using a vectorial analysis of the different axes with a computerised acquisition system. During the experiments, birds were placed unrestrained in the metabolic chamber and left to equilibrate for 1 h at the highest temperature tested for each age before metabolic rate and integrated shivering was monitored over 20 min. This initial period allowed a decrease and stabilization of Tb and MR at low resting values, indicating that the initial stress resulting from handling and confinement in the respirometer was largely reduced, if not abolished. The ambient temperature (Ta) in the metabolic chamber was then reduced in a stepwise fashion, and after the 1.5 hours necessary to reach metabolic steady state, the recording procedure was repeated for each temperature. Relations between metabolic rate and Ta or shivering activity and Ta were expressed by two linear regressions lines [19] that intersect at the lower critical temperature (LCT) or at the shivering threshold temperature (STT), as previously described [17,18]. Therefore, the LCT was determined as the Ta eliciting the first increase in metabolic rate, while STT was determined as the Ta eliciting the first increase in shivering activity.

### Muscle histochemistry

Pectoralis muscle samples from 1, 7, 15 and 30 day- old chicks were frozen in isopentane chilled with liquid nitrogen. A 5-mm-thick block from the mid portion of each muscle was mounted in embedding medium (TEK OCT. compound, Labonord, Paris, France) and stored at -80°C. Serial transverse sections (10µm) were cut on a cryostat and stained for myosin adenosine triphosphatase (mATPase) reactivity under pre-incubation conditions determined in Adélie penguin at pH 4.25, 4.45 and pH 10.3. Based on observed differences in pH lability of the myosin ATPase activity of the isomyosins in the different fibres, muscle fibres were classified into three major types, type I (slow-twitch, oxidative), type IIa (fast-twitch, oxidative-glycolytic) and type IIb (fast-twitch, glycolytic) and intermediate fiber types (int I or IIc and int II or IIab). Reduced nicotine amide adenine dinucleotide tetrazolium reductase (NADH-TR) activity was also shown at the different steps of pectoralis muscle development [20]. Distribution of fiber types was expressed as the number of fibres of each type relative to the total number of fibres per section. Measurements and analysis were made on a minimum of 500 fibres with a computerized planimetry system coupled with a digitizer (Visioscan software, Biocom, Paris, France).

### Data analysis

The relationship between shivering activity and ambient temperature, or metabolic rate and ambient temperature were expressed as two linear regression lines [17–19] that intersect at the shivering threshold temperature (STT) or at the lower critical temperature (LCT), respectively. To draw these regression lines, we statistically determined by a repeated measures analysis of variance (ANOVA) test at which ambient temperature shivering activity or metabolic rate became significantly different from basal values, respectively. These values and those measured at lower ambient temperature were then integrated in a second linear regression line distinct from the basal linear regression line, as previously used in birds [17,18]. Data are expressed as means ± S.E.M. Statistical differences were evaluated using a Kruskal-Wallis Test (StatView program). A Mann Whitney post hoc test was used for group comparison. A p-value < 0.05 was considered statistically significant.

## Results

### Body mass dynamics

Body mass data for growing Adélie penguin chicks were in the same range as those previously reported [8,21]. We therefore expressed their relative growth rate as the percentage of body mass gained per day, according to a commonly used calculation [8]. Chick body mass increased more than 40-fold over the first two months after hatching and 80% of the fledging body mass was achieved by 30 days of age. The relative growth rate then gradually decreased (Table 2). The surface area was estimated from body mass (S = 0.077 × mass^^2/3^^) [22]. As a consequence of body growth, the chick’s surface area increased more than 12- fold from D1 to D60, while its surface-to-volume ratio decreased markedly (-70%) during the first month and then remained stable (Table 2). These biological parameters with the concomitant increase in down thickness (Table 2) converged towards better insulation and a more favourable heat loss/heat production ratio.

**Table 2:** Morphological data.

	Post-hatching age (days)							
	1		7		15	30	60	
Chick status	Brooded		Partial cold exposure			Continuous cold exposure		
		Ectotherm			⇨	Endotherm				
Body mass (kg)	0.087±0.001		0.427±0.015		1.116±0.082	3.023±0.08	3.670±0.03	
	n=12		n = 12		n=12	n=12	n=12	
Surface area (m^2^)	0.015±0.001		0.041±0.001		0.082±0.004	0.161±0.002	0.183±0.001	
S/V (cm^-1^)	1.736±0.011		1.046±0.013		0.750±0.017	0.533±0.004	0.499±0.001	
Mean down length (mm)	7.2±0.7		11.3±0.2		15.6±0.7	36.3±1.2	_	
Relative growth rate (% gain mass /day)			13.2		7.7	4.2	0.5	

Changes in body mass, surface area, surface-to-volume ratio (S/V), mean down length and relative growth rate in growing Adélie penguin chicks. Values are means ± S.E.M. Surface area was estimated from body mass (S = 0.077 × mass^^2/3^^) Relative growth rate was estimated by calculating the percentage of mass gained per day.

### Developmental expression of key genes and proteins involved in pectoralis muscle maturation

**Figure 1:**
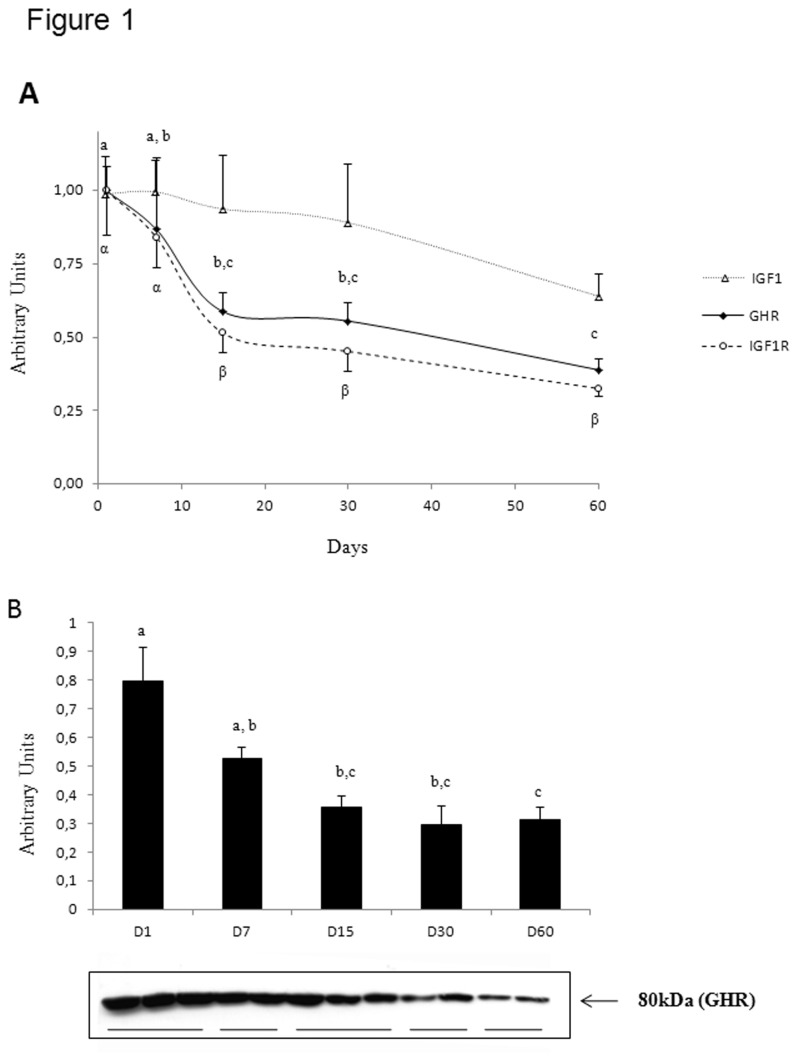
qPCR analysis of the expression of genes involved in growth. Panel A – qRT-PCR analysis of key genes involved in the control of growth, [Insulin-like Growth Factor (IGF-1), its receptor (IGF-1R) and growth hormone receptor (GHR)] in pectoralis muscle from 1, 7, 15, 30 and 60 –day- old Adélie chicks. The relative expression of each gene was expressed as a ratio to the β-actin mRNA level. Panel B - Protein abundance of growth hormone receptor (GHR) from the same chicks. N = 6 per group. Bars correspond to means ± S.E.M. values. Bars with different letters are significantly different at p<0.05.

To understand the molecular basis underlying the fast development and maturation of pectoralis muscle and the shift from ectothermy to endothermy, we first analysed the muscular expression of avGHR and avIGF-1R mRNA levels in 1 to 60 day- old Adélie chicks. The same developmental profile was observed for the two genes, with a sharp decline post hatching, during the nestling phase from D1 to D15, (-42% and -48% respectively; p<0.05). From D15 to D60, their relative expressions remained low and stable (Figure 1A). The kinetics of avGHR expression was confirmed by Western blot analysis, showing a significant decrease in protein levels during the nestling phase (-56%; p<0.05) (Figure 1B). In contrast, IGF-1 mRNA levels remained stable during the first 30 days and then decreased slightly but not significantly to 60 days (Figure 1A).

**Figure 2:**
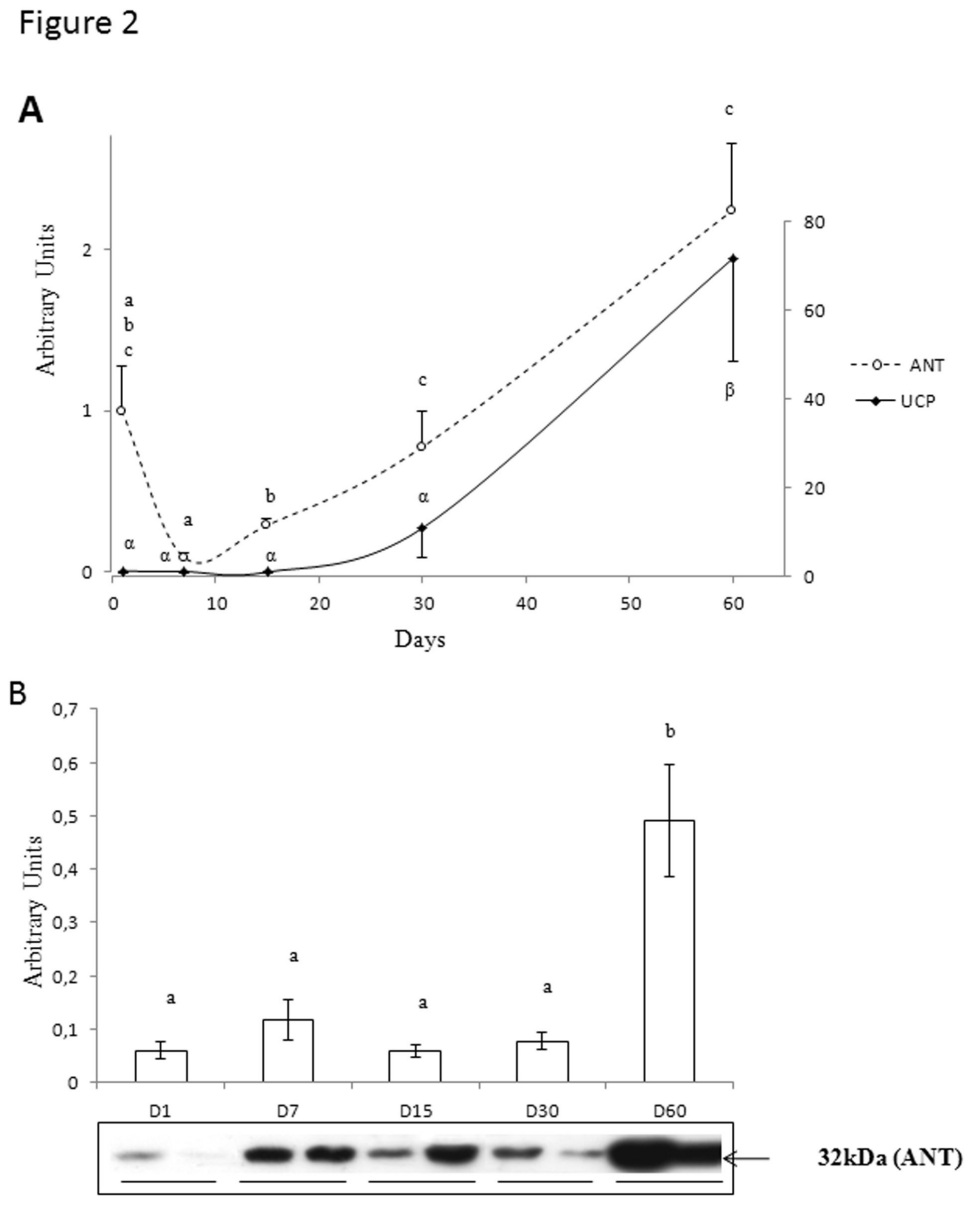
qPCR analysis of the expression of genes involved in mitochondrial energetic metabolism. Panel A – Progressive increases in the expression of mRNAs for avian uncoupling protein (avUCP) and avian adenine nucleotide translocase (avANT) in pectoralis muscle of 7, 15, 30 and 60 –day- old Adélie chicks. The relative expression of each gene was expressed as a ratio to β-actin mRNA level. Panel B - Protein abundance of avANT from the same chicks. N = 6 per group. Bars correspond to means ± S.E.M. Bars with different letters are significantly different at p<0.05.

The relative expression of mitochondrial avUCP and avANT genes remained very low and stable until D15 and then increased progressively to reach their maximum levels by D60 (Figure 2A). This developmental increase was confirmed at the protein level for avANT, as a sharp rise was observed between D30 and D60 (+532%; p<0.05) (Figure 2B).

**Figure 3:**
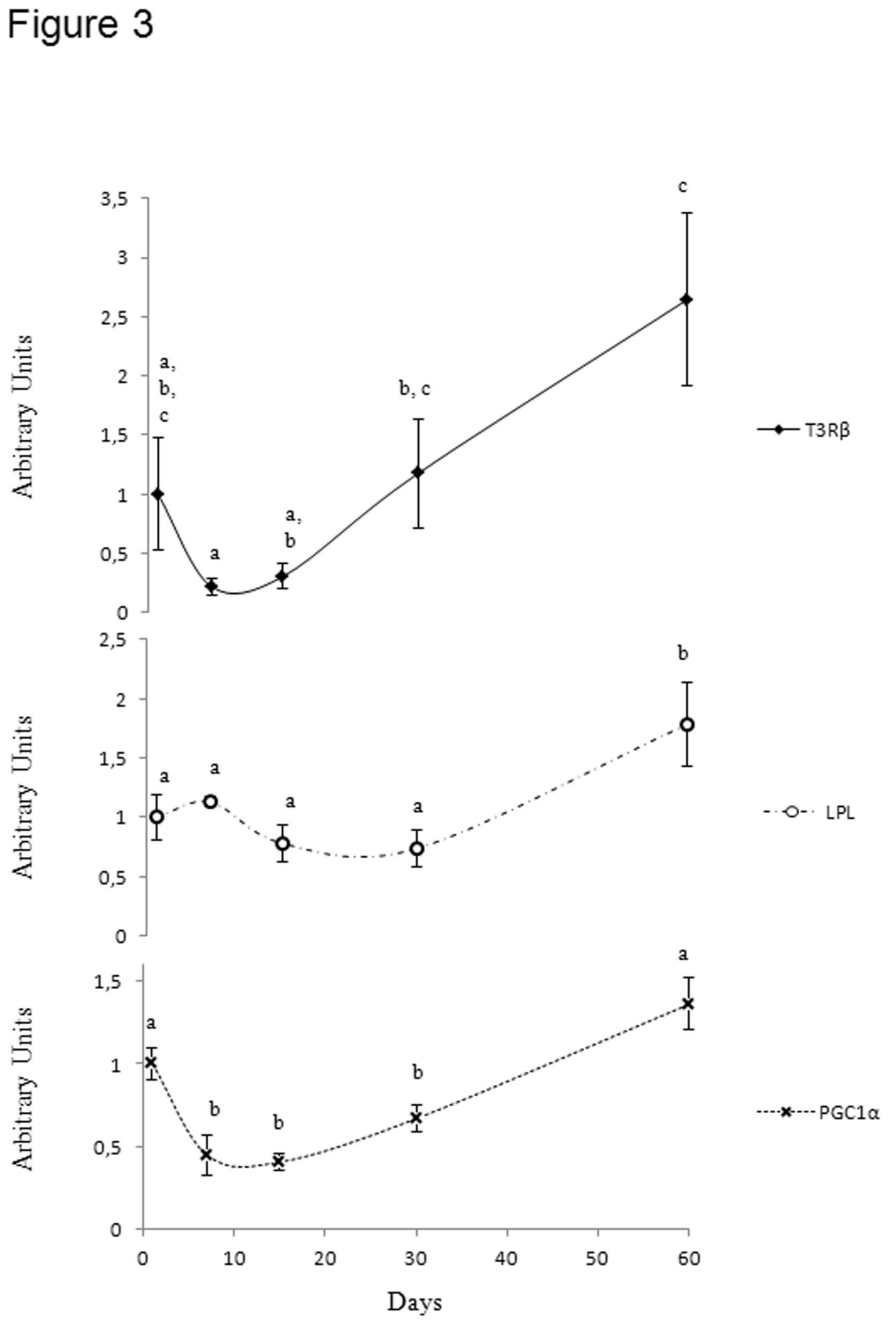
qPCR analysis of the expression of genes involved in transcriptional regulation and lipid metabolism. Time course of the levels of triiodothyronine receptor (T3Rβ), lipoprotein lipase (LPL), peroxisome proliferator-activated receptor-γ co-activator-1α (PGC-1α) transcripts in the pectoralis muscle of 1, 7, 15, 30 and 60-day- old Adélie chicks. The relative expression of each gene was expressed as a ratio to β-actin mRNA level. N = 6 per group. Bars correspond to means ± S.E.M. Bars with different letters are significantly different at p<0.05.

Concomitant with this change was a strong increase in avLPL mRNA levels (+141% from D30 to D60 p<0.05) (Figure 3), suggesting that muscle maturation requires lipid metabolism.

In addition to its role in growth [23], thyroid status and particularly triiodothyronine (T3) are considered as major regulators of energy metabolism and mitochondrial functioning, after binding to nuclear and mitochondrial T3 receptors [24]. As seen in Figure 3, T3Rβ gene expression increased markedly from D15 to D60 (11-fold; p<0.05) (Figure 3). As PGC-1α is also known to activate and coordinate gene expression that stimulates mitochondrial oxidative metabolism in the brown fat of rodents [25], and because its expression increases in the skeletal muscle of cold-exposed chickens, playing a crucial role in fibre specialization [26–28], we examined the involvement of avPGC-1α in pectoralis transformation. The level of avPGC-1α mRNA increased consistently from D15 to D60 (+235%; p<0.05) (Figure 3).

### Histochemical analysis of the developmental changes in pectoralis muscle oxidative capacity

**Figure 4:**
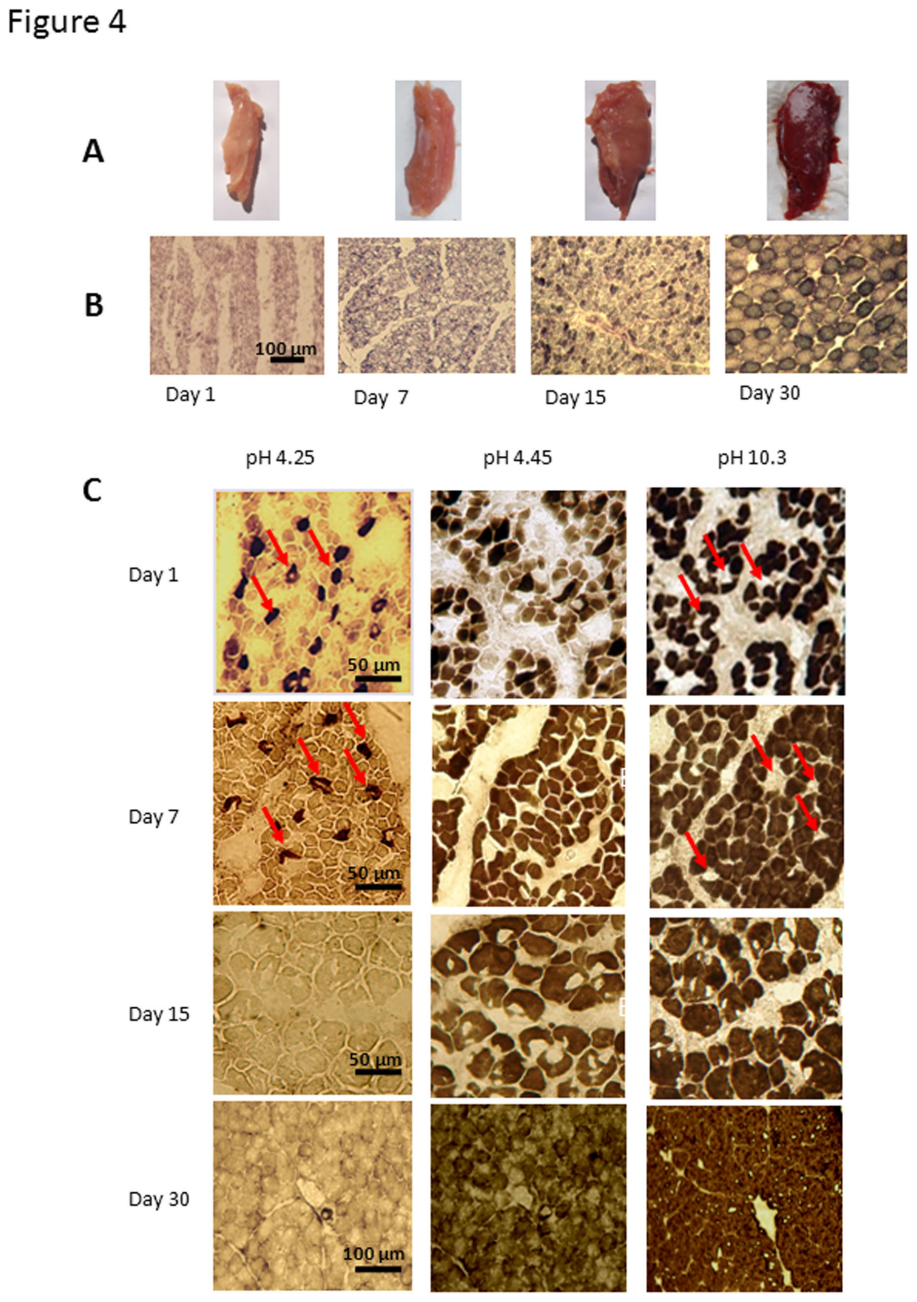
Morphological and histochemical properties of pectoralis muscle from 1 to 30 day -old Adélie chicks. Panels A - Pectoralis muscles showed a progressive red colour from hatching on D1 to D30. Panels B - The diameter and number of myofibers exhibiting strong NADH-TR (nicotine amide adenine dinucleotide tetrazolium reductase) activity increased with age. Panel C: Myosin ATPase reactions after preincubation at pH 4.25, 4.45 and 10.3, on pectoralis muscles from 1, 7, 15 and 30 -day- old Adélie chicks. Arrowheads show type 1 fibres.

A morphological examination of pectoralis muscle samples from 1 to 30 day- old chicks showed a progressively heightened red colouration that is characteristic of muscles possessing an oxidative form of metabolism and increasing myoglobin content (Figure 4A). Consistent with this morphological change, the number of myofibers exhibiting strong NADH-TR activity increased markedly in muscle sections from D15 chicks and signalled the acquisition of higher oxidative capacities (Figure 4B). Myosin-ATPase reactions further revealed a percentage of dark slow fibres, probably type I myofibers, reactive for acid-incubated mATPase, in the pectoralis muscle of chicks during the first stages of development (10.8± 2.5% at day 1 and 9.5 ± 2.2% at day 7; means obtained from 500 fibres, n=3 chicks/age). This fibre type disappeared in the pectoralis muscle of 15 day- old chicks, which was entirely composed of fast oxidative fibres (FOG) reactive for alkaline-incubated mATPase, but not for acid-incubated mATPase (Figure 4C).

### Metabolic response to cold

**Figure 5:**
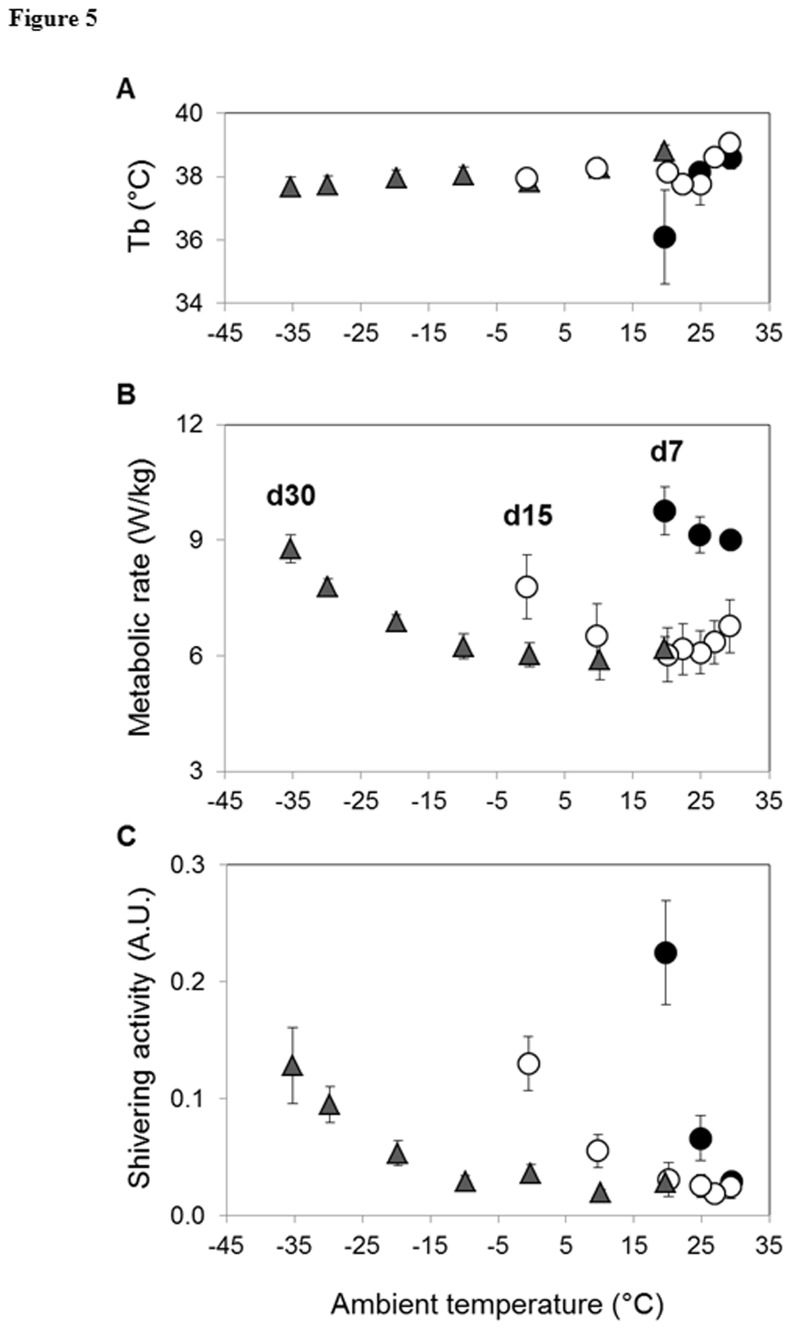
Analysis of whole animal metabolism response to cold. Relationship between body temperature (panel A), whole-animal metabolic rate (panel B), shivering activity (panel C) and ambient temperature in 7, 15 and 30-day- old Adélie penguins. Values are means ± S.E.M. from 9–12 individuals per age. When not visible, error bars are within the symbols. Symbols are as follows: D7, black circles; D15, white circles; D30, grey triangles.

LCT significantly decreased as Adélie penguin chicks grew up (Table 3), being negatively correlated with both body weight (LCT = 26.2 - 12.9 × BW, r = 0.96, p < 0.0001) and age (LCT = 37.5 - 1.6 × age, r = 0.98, p < 0.0001). At temperatures above LCT, RMR remained low and constant within a Ta range known as the thermoneutral zone. Because of the concomitant increase in body mass, the absolute resting energy expenditure expressed in watts per animal increased with age (Table 3). In contrast, the mass-specific RMR t started very high in 7-day- old chicks (8.5 ± 0.2 W/kg) before decreasing during the second week to reach a stable plateau at 15 days post-hatching (6.0 ± 0.7 W/kg at day 15 and 5.8 ± 0.3 W/kg at day 30) (Figure 5). Body temperature within the thermoneutral zone did not differ among groups (Table 3). The average respiratory quotient (RQ) was high in 7 and 15-day-old chicks, indicating their reliance on mixed substrates to fuel their metabolic activity. In 30 day-old chicks, the RQ dropped down to 0.75, suggesting a metabolic shift towards a greater reliance on lipid catabolism (Table 3).

**Table 3:** Critical temperatures and resting metabolic rates in growing Adélie penguin chicks.

	Post-hatching age (days)		
	7	15	30
Body mass (kg)	0.32 ± 0.01	0.71 ± 0.02*	2.9 ± 0.1*†
RMR (W)	2.7 ± 0.1	4.2 ± 0.6	16.6 ± 1.0*†
LCT (°C)	25.8 ± 1.0	13.7 ± 1.5*	-11.3 ± 1.3*†
STT (°C)	> 25	13.2 ± 1.7	-13.9 ± 1.4†
Tb in TNZ (°C)	38.2 ± 0.3	38.4 ± 0.1	38.3 ± 0.1
C (W/kg. ° C)	0.34 ± 0.04	0.13 ± 0.01*	0.12 ± 0.02*
RQ	0.87 ± 0.01	0.84 ± 0.01	0.75 ± 0.01*†

Changes in body mass, resting metabolic rates (RMR), lower critical temperature (LCT), shivering threshold temperature (STT), body temperature in thermoneutral zone (Tb in TNZ), thermal conductance (C) and respiratory quotient in 7 to 30 - day-old Adélie penguin chicks. Values are means ± S.E.M. from 9–12 individuals per age. *p<0.05, significantly different from D7; † p<0.05, significantly different from D15.

At temperatures below the LCT, there was a significant linear increase in metabolic rate as the Ta decreased (Figure 5). In response to cold exposure, poorly insulated 7-day-old chicks became hypothermic, suggesting a limited capacity for regulatory thermogenesis. Adélie penguins attain full endothermy from the age of 15 days, thus able to maintain their Tb in the cold (Figure 5). Shivering activity was simultaneously monitored with metabolic rate to determine the onset of shivering (Figure 5). As described for metabolic rate, the relationship between shivering activity and Ta was expressed as two linear regression lines that intersect at the STT. Above this threshold temperature, shivering activity was minimal and constant while, below this critical temperature, shivering activity increased linearly as Ta decreased. STT decreased as penguins grew older, and it was not significantly different from LCT in 7 and 15-day-old chicks (Table 3). This observation suggests that shivering is the only thermogenic mechanism present in growing Adélie penguin chicks during the first two weeks of life. STT became significantly different from LCT in 30-day-old chicks (Table 3), indicating that within this Ta range, regulatory thermogenesis was independent of shivering. However, at 30 days of age, penguin chicks displayed a small NST capacity of 0.4 ± 0.2 W/kg (i.e. 7% above RMR).

## Discussion

Our data show that in Adélie penguin chicks, from hatching to full independence, pectoralis muscle development is associated with a switch in gene expression that first underpins early and fast growth and then the metabolic changes inherent to its typical altricial/semi-altricial trajectory. These molecular events probably involve hormonally triggered transcription cascades and directly parallel an increased capacity for skeletal muscle oxidative metabolism.

### 1/ Fast growth in polar environments, why and how?

Adélie chick growth is remarkably rapid as body mass increases five-fold over the first six days and more than 20-fold within the first month of life. As previously reported [8], this intensive growth results from intensive parental care, which involves feeding the chick with large quantities of krill while it is protected from the cold. A maximal amount of energy can thus be diverted to growth and fat storage [9] rather than being dissipated as heat for thermoregulatory purposes. We had previously reported that skeletal muscle mass increases sharply with age [9]. As shown here, the pectoralis muscle exhibits the greatest mass increase (80-fold during the first month) compared to the quadriceps and gastrocnemius muscles (30 and 25-fold, respectively). In addition, it has been described as the main thermogenic tissue and its development and maturation are of major importance for the emancipation and survival of polar birds [12]. This muscle was therefore chosen as the key tissue for our molecular investigation. In contrast to chickens, which show a gradual increase in avGHR mRNA expression during the first week after hatching [16,29], our present data show a decline in the expression of pectoralis avGHR and avIGF-IR mRNA during the first week and up to 60 days after hatching. This might be surprising, as tissue sensitivity to growth factors generally depends on the abundance of its own receptors. Furthermore, GH concentration has been positively shown to control the expression of its receptor in avian skeletal muscle satellite cell cultures [14]. However, at the whole organism level, an apparent inverse relationship between plasma GH levels and avGHR expression is evident from ontogeny studies of commercial broiler chickens [15,16,30]. Although we were unable to access plasma pulsatile GH concentrations in Adélie chicks, it is therefore tempting to speculate that the rapid decline in avGHR expression corresponds to the sharp rise in plasma GH, which generally starts immediately before hatching in domestic birds [15]. The down- regulation of IGF-1R mRNA levels, however, is associated with a high and constant avIGF-1 mRNA expression in pectoralis muscle. Altogether, this suggests that the high and constant paracrine synthesis of avIGF-1 during the entire growth period of the chick may regulate muscle growth in a localised manner. This is further supported by the well-known role of extra-hepatic avIGF-1 in promoting muscle growth and protein synthesis [31,32]. Indeed, IGF-1 directly increases the proliferation of muscle cells isolated from chicken [33] and turkey [34]. Interestingly, increased avIGF-1 mRNA expression has also been reported in the pectoralis muscle of white-throated sparrows in “pre-migratory” condition. This molecular change was accompanied by increased flight muscle mass and elevated mass-specific oxidative capacity [35]. From these data, we suggest that, up to D11-15, the high postnatal growth rate of the Adélie chick (Table 2) [8] is supported by high levels of avGHR and avIGF-1R transcription, while the subsequent progressive down-regulation of these receptors would, in turn, suggest high levels of circulating hormones. On the whole, it appears that in the Adélie chick, early stimulation of the GH/IGF-1 axis enables a rapid and optimal growth of muscles. In addition, the constantly high levels of avIGF-1 mRNA observed in pectoralis muscle may be an important paracrine mediator of muscle growth and phenotypic maturation in anticipation of the energetic stress associated with the complete emancipation of Adélie penguin chicks, as reported in pre-migratory white-throated sparrows [35]. Finally, thyroid hormones may play an important role in this regulation. This is suggested by the high concentration levels of T3 found in our previous study during the early nestling period of Adélie chicks [9], by the fact that T3 replacement therapy partially restores growth in hypophysectomized chicks [23] and that hypothyroid chickens have a lower growth rate and lower IGF-1 production [36]. It has also been reported that circulating T3 influences circulating IGF-I bioactivity and local IGF-1 production in a tissue- specific manner [37]. In this case, the muscle expression profile of T3Rβ mRNA levels suggests that circulating T3 could play an important role in the late maturation of skeletal muscle, at least in part, through the reinforcement of the paracrine action of IGF-1.

### *2*/ Acquisition of endothermy, a prerequisite to survival in the cold

The development of endothermy requires both thermogenic capacity and insulation. Previous studies showed an increase in resting metabolic rate from hatching to D11 [8], therefore indicating an improvement in thermoregulatory processes [22]. However, during the nestling period (up to D7), chicks are unable to regulate their body temperature at a high and constant level, despite a significant shivering activity (Table 3 and Figure 5). The high surface-to-volume ratio and poor down insulation favour heat loss and could explain the inefficiency of shivering thermogenesis to maintain body temperature at 38°C. This implies that during the early post-hatching period, chicks have to be in close contact with the featherless brooding pouch of their parents in order to maintain a high body temperature. The consecutive reduction in energetic cost could probably favour a high growth rate by directing energy resources to tissue and body growth rather than to heat production. From D15, chicks acquire endothermic capacities as shown by their ability to maintain a high and constant body temperature over a wide range of cold ambient temperatures. At D30, shivering remains by far the main thermogenic process. These data clearly indicate that during the first month, shivering thermogenesis is the primary mode of regulatory thermogenesis, but that this thermogenic process only becomes efficient from D15 onwards, after thermal insulation has improved sufficiently due to body size (decreased area-to-volume ratio), down thickness and subcutaneous fat deposition [9]. It is reasonable to hypothesize that the ontogeny of endothermy is related to the morphology and development of the biochemical capabilities of pectoralis muscle. Based on the histochemical fibre-typing techniques used in this study, all fibres of the pectoralis muscle exhibit a fast-twitch, oxidative-glycolytic (FOG) phenotype from D15 onwards. This muscle phenotype is currently associated with an increase in mitochondrial content and a high substrate oxidation rate [38]. The predominance of FOG in pectoralis muscle is consistent with published studies on other birds that link intense metabolic demand for flight to the aerobic capability of muscle. For example, ontogenic increases in pectoralis muscle mass was found to be associated with abrupt biochemical changes that occur several days before the development of flight in the red-winged blackbird and it was stated that the composition of FOG fibres coincides with increases in aerobic, β-oxidative and myofibrillar ATPase activities [13]. Our results do not tell us whether these significant increases in pectoralis muscle mass during the first 30 days post-hatching are due to an increase in the number of fibres or simply to their hypertrophy. Notwithstanding, the disproportionate growth and the large increase in fibre size, probably supported by the persistence of muscular IGF-1 synthesis throughout the Adélie chick’s growth, increase the volume of this thermogenic tissue, thereby enhancing its thermogenic capacity. This muscular maturation is under the control of endogenous hormonal rhythms, as suggested by the elevated expression of circulating T3 and the up-regulation of tissue T3Rβ from D15 onwards. In addition to its role in growth, T3 is indeed considered to be a major regulator of energy metabolism and mitochondrial biogenesis in both mammalian and avian species [24]. As suggested by previous studies [25–28], the transcriptional co-activator avPGC-1α could also be a major regulator in the ectothermy/endothermy transition, in part by increasing the number of FOG fibres in the pectoralis muscle of cold exposed chickens. The increased level of avPGC-1α mRNA found in pectoralis muscle from D15 to D60 (Figure 3) would lead to the fibre specialization responsible for efficient thermogenesis in penguin chicks. Furthermore, previous studies have shown that cold exposure increases plasma T3 concentrations, which stimulates avian adenine nucleotide translocase (avANT) and avian uncoupling protein (avUCP) expression, directly or indirectly through the up-regulation of PGC-1α expression [28,39,40]. Our results reveal that avUCP and avANT concomitantly increase from D30 to reach high levels at D60. The late increase in avANT mRNA and protein abundance indicates a stimulation of muscular oxidative metabolism in response to extra ATP demands in the cytosol [41]. In addition, the change of fuel selection strategy, from carbohydrates at D7 and D15 to lipids at D30 and the up-regulation of avUCP, which may favour fatty acid oxidation through still undefined mechanisms [42], suggest that the pectoralis muscle undergoes profound metabolic changes in order to principally use lipids, i.e. energetic substrates that provide the highest ATP yield per gram of reserve [43]. Interestingly, lipids become the main energetic substrates at one month of age, once Adélie chicks display large functional mature adipocytes [9] and have almost completed their growth (Table 3). The markedly increased mRNA levels of muscle LPL at D60 further point to a higher capacity of skeletal muscle to extract fatty acid from circulating lipids.

In addition, avANT and avUCP are also able to increase mitochondrial inner membrane conductance in the presence of fatty acids or when activated by fatty acids and radical oxygen species [41,44–46], respectively. Cold-induced mitochondrial avANT expression has been associated with an increase in fatty acid dependent uncoupling of oxidative phosphorylation in skeletal muscle mitochondria of several bird species, including duckling, chicken and king penguin [41,44,46,47]. Similarly, the cold-induced up-regulation of skeletal muscle avUCP has been correlated with a higher avUCP activity and related increased mitochondrial inner membrane conductance in both penguins and ducklings [44,45] and associated with a higher thermogenic capacity of skeletal muscle [18]. Even though the thermogenic function of avUCP in skeletal muscle remains controversial, our data suggest that in Adélie penguin chicks, the up-regulation of avANT, avUCP and LPL, in association with the activation of lipid metabolism, altogether favour a fatty acid-enhanced thermogenesis by loose coupling mitochondrial oxidative phosphorylation at D60. Finally, the late up regulation of avUCP could play a role in slowing chick growth as demonstrated in transgenic experiments in mammals which show that a high level of UCP expression in skeletal muscle reduces the adult mass of transgenic mice despite increases in food supply [48].

This study provides a new perspective on the trade-off governing the postnatal development of Adélie penguin chicks by showing for the first time how molecular control allows their rapid growth and overcomes competitive energetic functions in a polar environment. The hierarchical transcriptional changes, associated with skeletal muscle development and differentiation, show how this species has managed the energetic challenge; first, it grows and then improves its thermoregulatory processes. This molecular pattern is probably not specific to Adélie penguin; it might apply to other altricial species facing similar environmental and temporal constraints. However, from morphological and metabolic comparative studies [4,49–51], it appears that each species has its own strategy for managing the energy costs of growth and functional maturity. There is even the suggestion that every muscle has its own rate of development according to its function (locomotion, flight, swimming) and the importance of this function for the rapid acquisition of autonomy by the chick. Further molecular comparative studies including the use of different muscles and more species could therefore improve our understanding of the antagonism between growth and functional maturity that restricts postnatal development.

In conclusion, the life-history trade-offs and their physiological causes and consequences continue to be a major issue in evolutionary biology but current paradigms do not seem to be sufficient to explain the diversity of patterns shown by wild animals. From the molecular level to the whole organism, integrative studies applied to phenotypic traits will provide a better understanding of how animals develop and function in their natural environment.

## Supporting Information

Figure S1 **EMG and accelerometry simultaneously recorded on ducklings exposed to 25°C (thermoneutrality) and 12°C (cold exposure).** Both signals are highly sensitive (detection of heart beat) and equally detect shivering activity.
(TIF)

Figure S2 **qPCR analysis of the expression of GHR and avUCP.** Variations in mRNA expression for growth hormone receptor (GHR – Panel A) and avian uncoupling protein (avUCP – Panel B) in pectoralis muscle from 1, 7, 15, 30 and 60-day- old Adélie chicks. The relative expression of each gene was expressed as a ratio to the 18S rRNA level. N = 6 per group. Bars correspond to means ± S.E.M. Bars with different letters are significantly different at p<0.05.
(TIF)
