# Supplementary figures and images for: Growth Prior to Thermogenesis for a Quick Fledging of Adélie Penguin Chicks (Pygoscelis adeliae)
Source: PLoS One. 2013 Sep 6;8(9):e74154. doi: 10.1371/journal.pone.0074154 (PMC3765356; doi:10.1371/journal.pone.0074154)

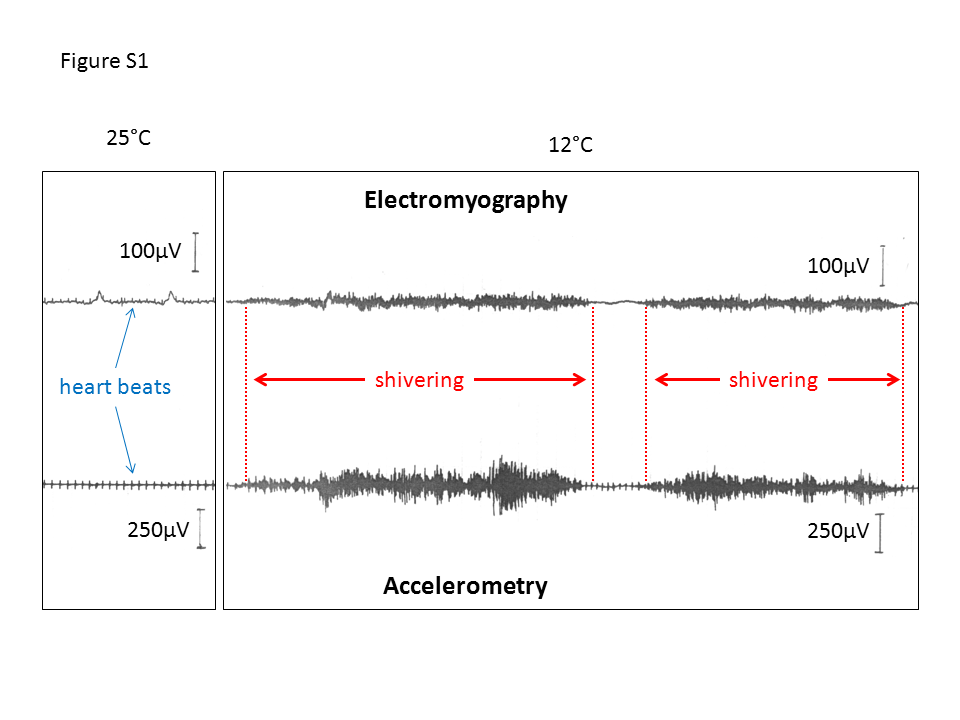

Supplement: Figure S1 — EMG and accelerometry simultaneously recorded on ducklings exposed to 25°C (thermoneutrality) and 12°C (cold exposure). Both signals are highly sensitive (detection of heart beat) and equally detect shivering activity. (TIF) [file pone.0074154.s001.tif]

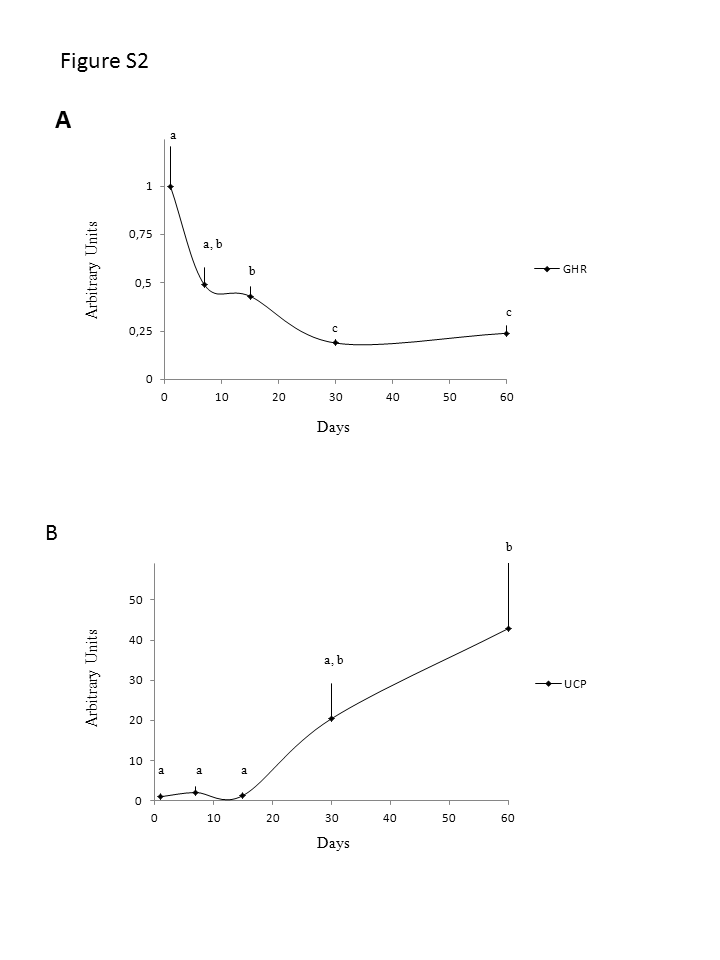

Supplement: Figure S2 — qPCR analysis of the expression of GHR and avUCP. Variations in mRNA expression for growth hormone receptor (GHR – Panel A) and avian uncoupling protein (avUCP – Panel B) in pectoralis muscle from 1, 7, 15, 30 and 60-day- old Adélie chicks. The relative expression of each gene was expressed as a ratio to the 18S rRNA level. N = 6 per group. Bars correspond to means ± S.E.M. Bars with different letters are significantly different at p<0.05. (TIF) [file pone.0074154.s002.tif]
